# Hardness Changes Due to the Morphological Evolution of Microstructural Phases in an As-Solidified Zn-Fe Alloy

**DOI:** 10.3390/ma18061311

**Published:** 2025-03-16

**Authors:** Guilherme Calixto Carneiro de Sousa, Andrei de Paula, Andre Barros, Amauri Garcia, Noé Cheung

**Affiliations:** Department of Manufacturing and Materials Engineering, University of Campinas—UNICAMP, Campinas 13083-860, SP, Brazil; g238685@dac.unicamp.br (G.C.C.d.S.); a248824@dac.unicamp.br (A.d.P.); amaurig@fem.unicamp.br (A.G.); cheung@fem.unicamp.br (N.C.)

**Keywords:** Zn alloys, microstructure, solidification, hardness

## Abstract

Zn-Fe alloys are gaining attention for their use as bioabsorbable implants, and their development requires a deeper understanding of the processing–microstructure–property relationships. This study aimed to analyze the influence of microstructural features on the hardness of a Zn-2 wt.%Fe alloy. To achieve this, a casting was fabricated using directional solidification, and samples that experienced various cooling conditions were extracted from it. The results show that the microstructure of the investigated alloy was composed of a Zn-rich phase (matrix) and FeZn_13_ intermetallic particles. Four different morphological patterns of the microstructure could be formed, depending on the thermal conditions during solidification. For each of these patterns, a reduction in the spacing between FeZn_13_ particles, a parameter representing the degree of microstructural refinement, did not lead to a considerable increase in the hardness of the Zn-2wt.%Fe alloy. Hardness was shown to be more dependent on the morphology of the FeZn_13_ intermetallics and Zn-rich matrix than on the degree of refinement of these microstructural phases. Therefore, the present research provides valuable insights into the development of enhanced Zn-Fe alloys by demonstrating how microstructural features can affect their properties, particularly in terms of hardness and morphologies of the microstructural phases.

## 1. Introduction

Over the past few decades, several studies on biodegradable metallic implants have primarily focused on two categories of materials: Fe- and Mg-based alloys [1]. In this context, Fe-based alloys exhibit good biocompatibility and superior mechanical strength; however, their corrosion rate can be slow, leading to prolonged implant retention, and the formation of thick iron oxide layers may induce inflammatory responses [2]. Mg-based alloys present more favorable biodegradation rates and biocompatibility, but their rapid corrosion can result in excessive hydrogen gas release, potentially causing tissue separation and gas embolism [3]. In view of these limitations, Zn has emerged as a promising base metal for biodegradable alloys, especially due to its essential role in physiological processes and the intermediate corrosion rate between Fe and Mg [4].

Although pure Zn exhibits insufficient mechanical strength for load-bearing applications, the addition of appropriate alloying elements can enhance its mechanical properties while maintaining excellent biocompatibility and suitable in vivo degradation behavior [5]. Particularly, Zn-based alloys are promising for the fabrication of bioabsorbable implants, such as stents and orthopedic fixation devices. Nevertheless, their development for biomedical applications can be considered a relatively recent research field [1]. Li et al. [6] report that several Zn-based alloys are commercially available; however, some, such as the ZA and Zamak series, contain Al, which is associated with adverse health effects, including neurotoxicity and a potential connection with Alzheimer’s disease [7]. To address these concerns, a promising strategy is to alloy Zn with biocompatible and essential elements [8].

Considering the potential cytotoxicity of certain alloying elements, such as Al, ensuring biocompatibility is an important consideration in the development of biodegradable Zn alloys. Among these elements, Fe is recognized as an attractive candidate since it is biodegradable and one of the most abundant transition metals in the human body. Hence, adding Fe to Zn is a strategic approach to enhance the mechanical and corrosion properties of Zn alloys. Fe also plays an important role in various biological functions, further improving the biocompatibility of Zn-based implants. As can be seen, Fe incorporation has positive impacts on the mechanical strength of Zn alloys while maintaining good biocompatibility, as its degradation products are metabolizable [9]. In an in vivo study, Kafri et al. [10] compared Zn-2wt.%Fe implants with inert Ti implants in rats. The results revealed a comparable biological response between the two groups. The higher corrosion rate observed in Zn-2wt.%Fe implants, rather than being a drawback, is a desirable characteristic for biodegradable biomaterials, as it promotes implant degradation and resorption within the body. In an in vivo evaluation, Kafri et al. [10] further demonstrated that rats implanted with Zn-2wt.%Fe exhibited a biological response consistent with a control group implanted with inert Ti-6Al-4V alloy. This suggests that the Zn-2wt.%Fe alloy has great potential for biomedical applications.

From a physical metallurgy perspective, regardless of whether traditional or advanced processing techniques are employed, the solidified metal serves as the raw material for subsequent mechanical processing steps, which are essential for shaping Zn-based alloys into specific biodegradable medical devices. A typical issue during the solidification of Zn-Fe-based alloys is the formation of coarse secondary phases, especially those alloys containing Fe, Ca, and Sr. This occurs due to the low solid solubility of these elements in Zn and the tendency of intermetallic compounds to solidify before the Zn-rich matrix [1]. For instance, in Zn-1.3wt.%Fe and Zn-3wt.%Cu-1wt.%Fe alloys, FeZn_13_ particles exceeding 50 µm and 100 µm in size, respectively, have been reported. Plus, in Zn-1wt.%Mn-0.1wt.%Fe alloys, (Fe,Mn)Zn_13_ needle-like structures can achieve lengths greater than 800 µm [5,11,12]. These coarse particles reduce the ductility of Zn-Mn-Fe alloys, especially as the Fe content increases. Furthermore, the thermal cooling conditions during solidification directly influence microstructural formation. However, the effect of these variables on the mechanical properties of the Zn-2wt.%Fe alloy remains barely understood.

Recent studies confirmed the impact of the microstructure on the properties of Zn-Fe-based alloys and highlighted their potential applications. Li et al. [13] investigated a biomedical Zn-Fe alloy fabricated using low-temperature sintering and reported that grain refinement reduced stress concentration and improved resistance to dislocation and crack growth, resulting in improved hardness and compression yield strength. They also found out that the formation of intermetallic FeZn_13_ phases contributed to precipitation strengthening, reinforcing the potential of Zn-Fe alloys for orthopedic implants. Su et al. [14] observed that the uniformly distributed secondary phase in Zn-Fe alloys enhanced the mechanical properties and favored uniform degradation, thereby improving biocompatibility, especially in the Zn-0.4Fe alloy. Zhang et al. [15] reported that Li addition to Zn-Fe alloys (prepared by melting and hot extrusion) had positive effects on the microstructure, mechanical properties, corrosion behavior, and biocompatibility. Among the compositions studied, the Zn-0.5Fe-0.5Li alloy demonstrated excellent corrosion behavior and the most favorable in vitro cytocompatibility in cellular experiments. It is worth mentioning that, according to Bhat et al. [16], Zn-Fe alloy coatings can be used in the automotive industry. Zhang and Yao [17] studied a biodegradable Zn-Fe coating with superhydrophobic properties applied to the surface of ultra-light Mg-Li alloy. They found that the coating reduced the degradation rate.

Based on these considerations, the main objective of this study is to investigate the microstructural evolution of the Zn-2wt.%Fe alloy produced by directional solidification and analyze its influence on the resulting Vickers hardness. The novelty of this study lies in the investigation of how solidification thermal parameters influence the microstructure–property relationship in the Zn-2wt.%Fe alloy. It is important to consider that liquid-to-solid transformation is widely common during the processing stages of manufacturing parts made of metallic alloys. Additionally, we explore how different microstructural morphologies can impact the mechanical behavior of the Zn-2wt.%Fe alloy in terms of hardness, a relatively simple property to analyze which strongly correlates with mechanical strength. Therefore, this work offers valuable insights into the development of Zn-Fe alloys with enhanced properties.

## 2. Materials and Methods

**Figure 1:**
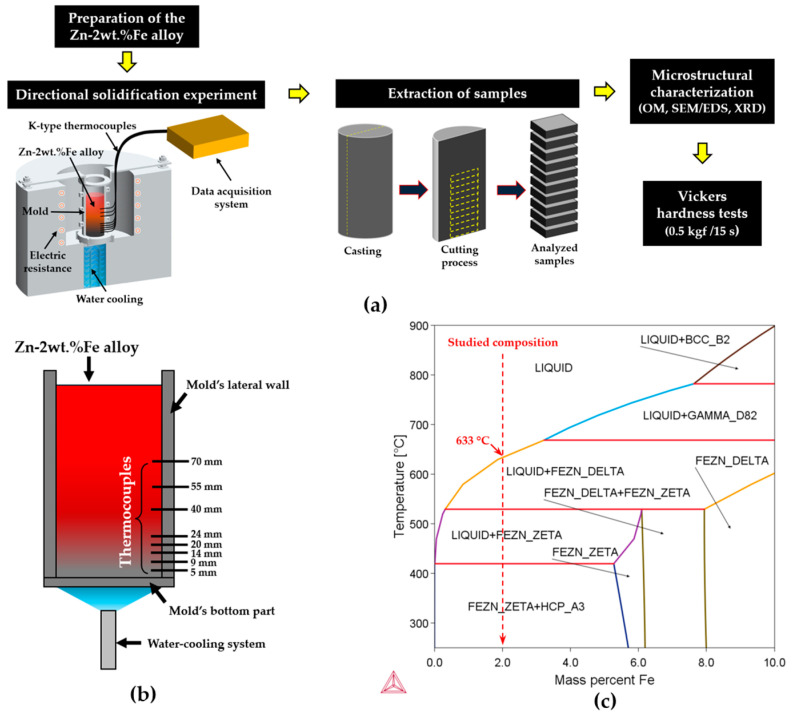
(**a**) Schematic sequence of the procedures followed in this study, (**b**) diagram indicating the temperature measurement positions during solidification, and (**c**) Zn-Fe phase diagram with indication of the studied composition.

Figure 1a shows a schematic flowchart of the experimental methodology employed in this study for the characterization of the Zn-2wt.%Fe alloy, Figure 1b shows a diagram indicating the temperature measurement positions during solidification, and Figure 1c shows the Zn-Fe phase diagram, indicating the studied composition. In Figure 1a, the steps include alloy preparation, directional solidification experiment, extraction of samples from the produced casting, microstructural characterization, and hardness testing.

For the fabrication of the Zn-2wt.%Fe alloy samples, approximately 2 kg of commercial-grade zinc was first prepared and placed in a SiC crucible, which was heated in a muffle furnace to 750 °C. This temperature was set to ensure that the alloy remained fully in the liquid state during its preparation, also considering a melt superheat of 10% above the liquidus temperature (T_L_), which is a common practice in studies involving directional solidification. As shown in the Zn-Fe phase diagram in Figure 1c, the value of T_L_ for this alloy was 633 °C. Adding 10% melt superheat resulted in 696.3 °C, which, when rounded, gave approximately 700 °C. A safety margin of 50 °C above this temperature was considered. The amount of 2 kg for Zn was chosen based on the dimensions of the mold used, which accommodated a volume of approximately 340 cm^3^. After the zinc was melted, the stoichiometric amount of commercial-grade Fe (99.9% purity) was added to the molten Zn, and the mixture was mechanically homogenized to ensure complete Fe dissolution.

Table 1 shows the chemical compositions of the commercially pure metals used. After homogenization, the molten material was poured into a cylindrical AISI 310 stainless-steel mold placed within the directional solidification device’s casting chamber. The mold, with an internal radius of 30 mm and a height of 150 mm, consisted of two semicylindrical shells fixed with M6 screws and nuts, along with an AISI 1020 carbon steel base. To facilitate the removal of the cast piece and avoid contamination, the internal walls of the mold were coated with an alumina-based refractory material. Furthermore, K-type thermocouples (Ø 1.6 mm) were installed in through-holes at different heights of the mold for temperature monitoring.

The directional solidification device used in this study had the advantage of promoting a wide range of transient cooling conditions throughout the length of the casting. With an appropriate cutting procedure, it was possible to extract samples with different microstructural length scales in a single experiment. The directional solidification experiment with the Zn-2wt.%Fe alloy aimed to achieve a broad range of cooling rates, resulting in different microstructures along the casting. The directional solidification device was equipped with lateral electrical resistances, which allowed reheating of the liquid metal after it was poured into the stainless-steel mold. K-type thermocouples were connected to a LynxADS1000 (Lynx, São Paulo, Brazil) data logger for real-time temperature monitoring, collecting data every 0.2 s. As soon as the thermocouple positioned closest to the base of the mold recorded a temperature approximately 1.1 times higher than the liquidus temperature (T_L_) of the Zn-2wt.%Fe alloy, the electrical resistances were deactivated, and the water flow at the base of the mold was activated to initiate the solidification process.

The thermal data obtained during the experiment were used in the calculation of cooling rates at the liquidus temperature (Ṫ_L_) and the solidification front velocity (V_L_). These thermal parameters were determined based on the temporal derivatives of the relationships between temperature and time, as well as between position and time, considering the moment when the liquidus isotherm reached the corresponding thermocouple. With these data, it was possible to determine the profiles of the thermal solidification parameters along the length of the casting, as in previous studies [18]. A second-order polynomial trend line was fitted to each cooling curve to represent the temperature–time behavior in a region around T_L_. The resulting equations were expressed in the following general form:

(1) $$T\left(t\right)=At^{2}+Bt+C$$

where T (t) = temperature at given time t and A, B, and C = coefficients.

Equation (1) was solved for each fitted temperature profile considering the case T(t) = T_L_, resulting in two values for t. The one closest to the experimentally measured time represented the time when the liquidus isotherm front passed (t_L_). Then, Ṫ_L_ was determined by taking the first-time derivative of Equation (1) and the value t_L_, as shown below.

(2) $$\frac{dT(t)}{dt}={\dot{T}}_{L}=2At+B$$

By associating each value of Ṫ_L_ with its corresponding relative thermocouple position (P), a set of ordered pairs was formed, (P_1_, Ṫ_L_(t_L1_)), (P_2_, Ṫ_L_(t_L2_)), …, (P_n_, Ṫ_L_(t_Ln_)), where n represented the number of thermocouples used in the experiment. Based on these data, an experimental power trend line was fitted to represent the Ṫ_L_ profile along the length of the casting, as shown in Equation (3):

(3) $${\dot{T}}_{L}\left(P\right)=aP^{-b}$$

where Ṫ_L_(P) = Ṫ_L_ at position P; a = constant; and b = exponent.

The association of each t_L_ value with its corresponding P value resulted in a set of ordered pairs, (t_L1_, P_1_), (t_L2_, P_2_), …, (t_Ln_, P_n_), where n represents the number of thermocouples used in the experiment. Based on these data, an experimental power trend line was determined to characterize the displacement of the liquidus isotherm along the length of the casting, as shown in Equation (4):

(4) $$P\left(t\right)=ct^{d}$$

where P(t) = position at time t; c = constant; and d = exponent.

By calculating the first-time derivative of Equation (4), V_L_ could be determined as a function of time, as represented in Equation (5).

(5) $$\frac{dP(t)}{dt}=V_{L}=cdt^{d-1}$$

By rearranging Equation (4) to isolate the variable t and substituting it into Equation (5), the term V_L_ could be expressed as a function of the relative position in the casting, as follows:

(6) $$V_{L}\left(P\right)=\frac{cd}{c^{\frac{d-1}{d}}}P^{\frac{d-1}{d}}={eP}^{-f}$$

where V_L_(P) = V_L_ at position P; e = constant; and f = exponent.

After the solidification process was completed, several samples were extracted from the casting by vertically cutting it into two similar semicylindrical parts. One of these parts was used for macrostructural analysis, being ground up to a #1200 grit and chemically etched with aqua regia reagent. The other part of the casting was used for extracting transverse samples, perpendicular to the solidification direction, for microstructural analysis. These samples were cut at specific intervals, ground with abrasives ranging from #100 to #1200 grit, and polished with diamond pastes from 3 µm to ¼ µm, using intermittent alcohol jets during polishing. After polishing, the samples were chemically etched with 3% Nital for approximately 10 s to reveal the microstructure.

Following metallographic preparation, the samples were analyzed using optical microscopy (OM) with an Olympus GX41 microscope (Olympus, Tokyo, Japan) to quantify microstructural spacings. Additionally, scanning electron microscopy and energy-dispersive X-ray spectroscopy (SEM/EDS) were performed using a ZEISS EVO MA 15 (ZEISS, Oberkochen, Germany) equipped with an Oxford-XMax EDS detector (Oxford Instruments, High Wycombe, UK). Furthermore, the samples were analyzed by X-ray diffraction (XRD) using a Philips Analytical X-Ray, X’Pert-MPD model, with copper Kα radiation (λ = 1.54056 Å). The XRD patterns acquired using a diffractometer with a 2θ range of 30° to 90° were analyzed by comparing them with crystallographic data from the Inorganic Crystal Structure Database (ICSD) to identify the phases present in the microstructure.

Vickers hardness measurements were carried out according to ASTM E384 using a Shimadzu HMV-2 device (Shimadzu, Kyoto, Japan). A load of 0.5 kgf was applied for 15 s. The Vickers hardness value for each sample was the average value of 25 indentations.

## 3. Results and Discussion

### 3.1. Zn-Fe Phase Diagram

Figure 1c shows the Zn-Fe phase diagram (obtained using the Thermo-Calc software version 2021b with its TCAL8 database (TCAL8: Al-Alloys v8.2 database), which reveals that the microstructure of the Zn-2wt.%Fe alloy primarily consists of a zinc matrix (HCP_A3) and FeZn_13_ intermetallic particles (FEZN_ZETA). This microstructure results from the low solubility of Fe in Zn in the solid state. FeZn_13_ is considered a highly relevant secondary phase. According to the phase diagram, solidification of the Zn-2wt.%Fe alloy begins with the formation of FeZn_13_ before the Zn matrix. Since an Fe atom bonds with thirteen Zn atoms to form the FeZn_13_ intermetallic compound, even a small addition of Fe can lead to a significant volume fraction of FeZn_13_ particles. According to Shi et al. [11], the formation of coarse secondary phases is a concern in Zn alloys containing elements such as Fe, Ca, and Sr. This is attributed to the low solubility of these elements in Zn and the tendency of intermetallic compounds to solidify before the Zn-rich matrix. In Zn-1.3wt.%Fe [5], Zn-3wt.%Cu-1wt.%Fe [10], Zn-2%Fe-(0.3 to 0.8%)Mn [19], and Zn-1wt.%Mn-0.1wt.%Fe [11] alloys, coarse FeZn_13_ particles have been observed, which negatively affect the material’s ductility. The thermal conditions for solidification have a significant impact on the morphology of FeZn_13_ particles, as will be shown in this work.

### 3.2. Solidification Microstructure and Thermal Parameters

**Figure 2:**
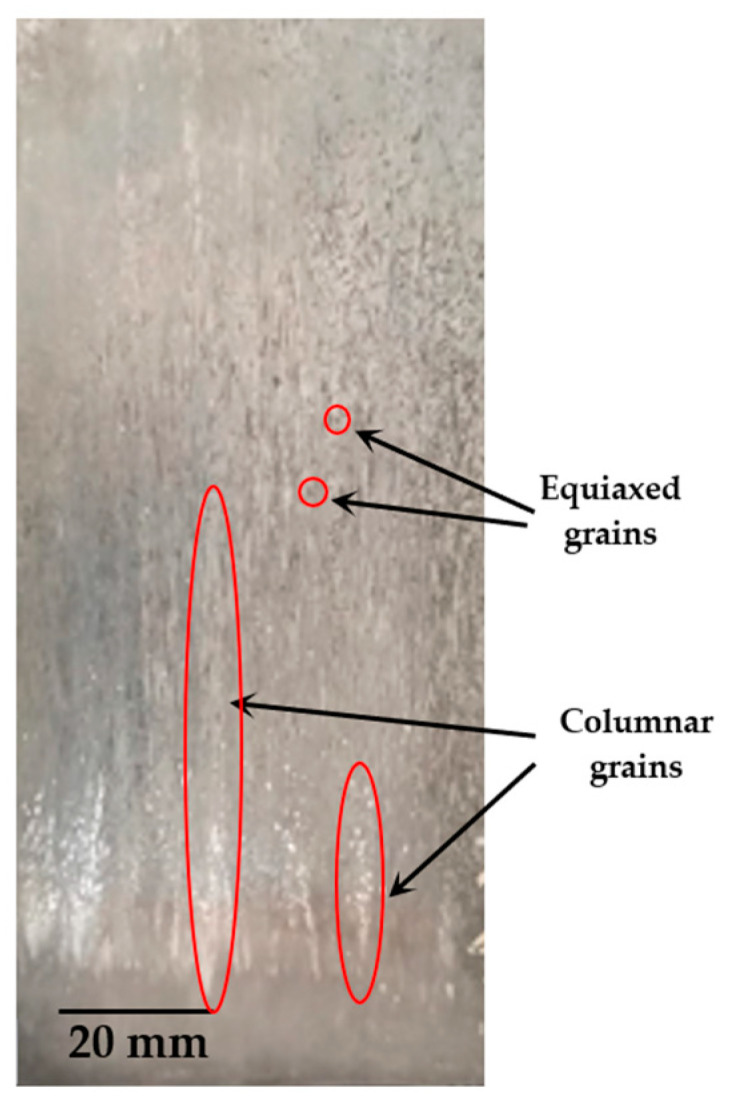
Macrostructure of the produced casting.

**Figure 3:**
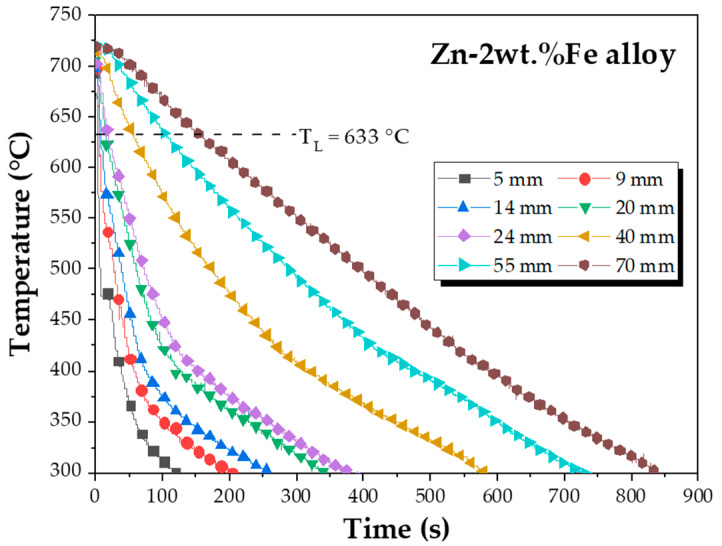
Experimental cooling curves.

The macrostructure of the produced casting is shown in Figure 2, where the coexistence of columnar and equiaxed grains is observed. A possible reason for this is the fact that, as the columnar structure develops, some equiaxed grains become trapped in the channels between the grains, while others sediment. This means that there is a competition between equiaxial nucleation and columnar growth, with the equiaxed grains formed not hindering the formation and growth of columnar dendrites [20]. Figure 3 shows the experimental cooling curves recorded along the length of the Zn-2wt.%Fe alloy casting. The position values shown in the figure refer to positions from the base of the casting, indicating heights. Overall, cooling curves obtained in regions closer to the base of the casting exhibited more pronounced temperature variations. Furthermore, as the height of the thermocouples increased, the intensity of these variations diminished. In other words, the directionally solidified casting experienced different thermal conditions along its length.

**Figure 4:**
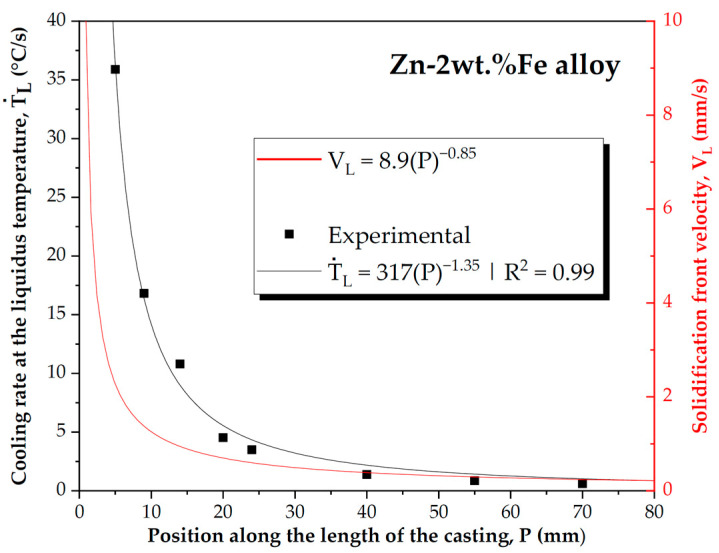
Ṫ_L_ vs. P and V_L_ vs. P curves.

The analysis of the cooling curves shown in Figure 3 allowed for the determination of the solidification thermal parameters Ṫ_L_ and V_L_, which are plotted in Figure 4 as a function of their position along the casting (P). Both thermal parameters exhibited higher values in regions close to the base of the casting, decreasing towards more distant positions. The occurrence of such decreasing profiles along the length of the casting was due to the increase in thermal resistance of the solidified layer and the thermal resistance at the metal–mold interface. To explain the decreasing profiles of the Ṫ_L_ vs. P and V_L_ vs. P curves, Fourier’s law could also be applied, stating that the rate of heat flow was inversely proportional to the thickness of the solidified layer. Thus, as the solid layer thickness increased, the rate of heat flow through it decreased. Given that the samples were extracted from the casting between P values of 5 and 70 mm, the Ṫ_L_ and V_L_ ranges considered for analysis spanned from 1.02 to 36.10 °C/s and from 0.24 to 2.27 mm/s, respectively.

### 3.3. Microstructural Evolution

**Figure 5:**
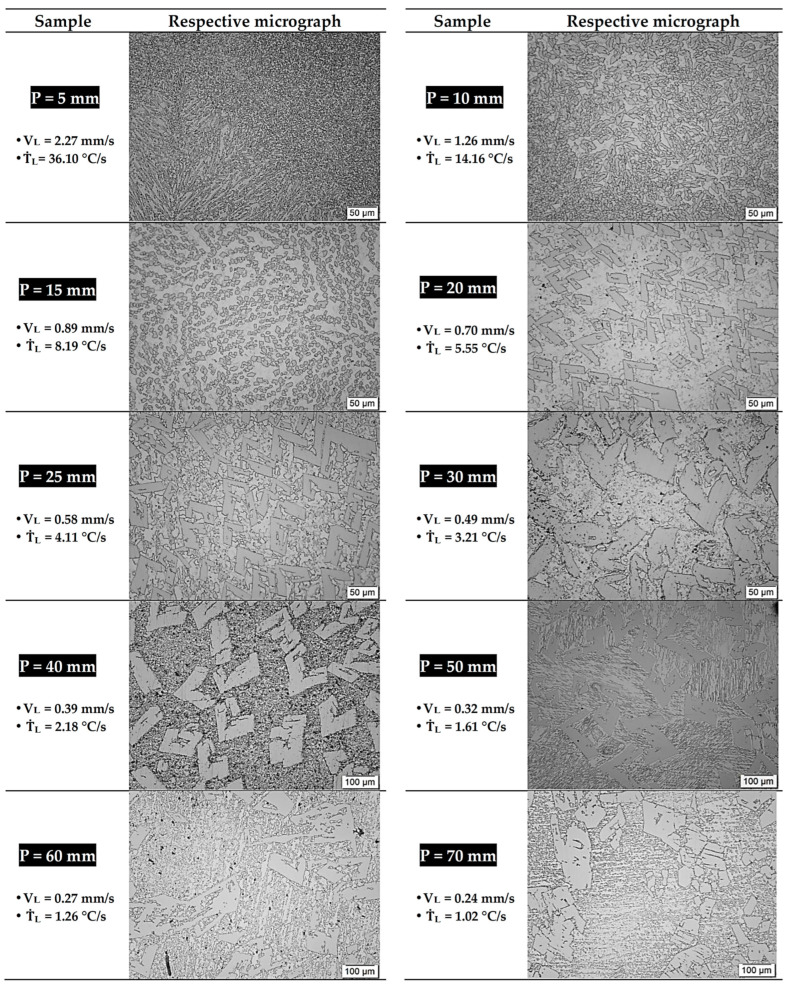
Characteristic micrographs observed along the length of the Zn-2wt.%Fe casting.

Figure 5 shows optical images of samples collected along the length of the Zn-2wt.%Fe alloy casting. These microstructures show similarities to those found in the studies by [5,8]. Based on the previously mentioned studies and the Zn-Fe phase diagram (Figure 2), it could be inferred that the microstructure consisted of FeZn_13_ particles distributed at the grain boundaries of a matrix predominantly rich in Zn. It could also be observed that the samples subjected to higher cooling rates exhibited finer microstructures, as expected. The variation in the solidification thermal parameters not only promoted changes in the microstructural length scale but also induced changes in the morphology of the Zn-rich and FeZn_13_ phases.

In regions with more intense cooling, the FeZn_13_ particles were finer, while, in areas with slower cooling, these particles became coarser. Furthermore, the morphology of these particles developed into complex microstructural patterns, resulting in four distinct microstructural patterns. For the samples corresponding to the positions of 5 to 10 mm along the length of the casting, a circular profile for the FeZn_13_ intermetallics was observed. At the 15 mm position, the first (1st) morphological transition occurred, with alignment of these intermediate particles. At the 20 mm position, the second (2nd) morphological transition began, with the formation of FeZn_13_ intermetallics with “V” or “L” profiles and the emergence of cellular morphology in the Zn-rich matrix. This profile persisted until the 40 mm position, differing only in the degree of microstructural refinement. Starting from the 50 mm position, the third (3rd) and final transition occurred, where a lamellar profile was identified in the Zn-rich matrix. This morphology persisted until the last position of the casting, with the difference being the increasingly complex shapes of the intermetallic profiles.

**Figure 6:**
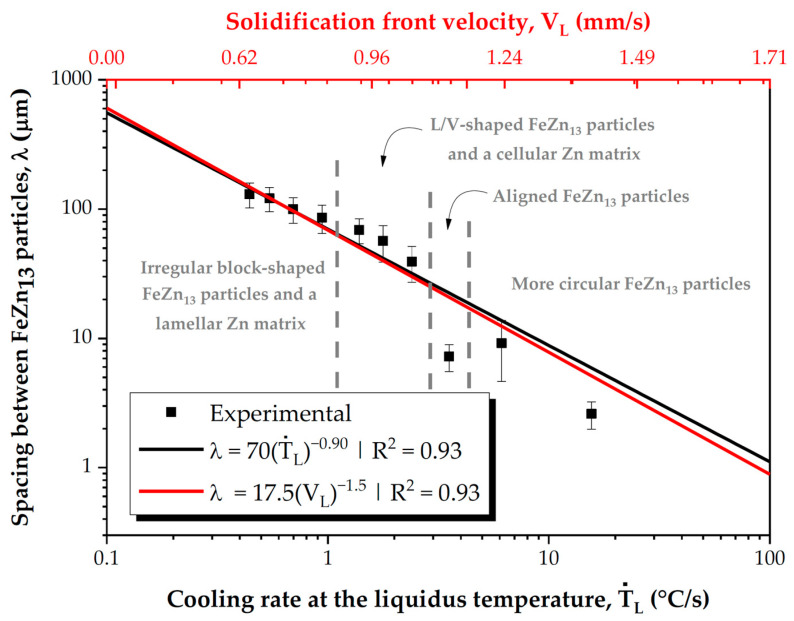
Relationship of the spacing of FeZn_13_ particles (λ) with Ṫ_L_ and V_L_. The dashed lines in the graph indicate the boundaries between different morphological patterns of the microstructural phases.

The quantitative analysis of the microstructure was performed by measuring the spacing between FeZn_13_ particles (λ) using the triangle method [21]. The results shown in Figure 6 demonstrate direct correlations between the microstructural parameter λ and both Ṫ_L_ and V_L_, which can be expressed through power law equations. It is important to note that the coefficient of determination (R^2^) calculated is a statistical measure that indicates how well the observed data fit a regression model. High values for R^2^ (>0.9) indicate a very good fit of the equations to the experimental data. It is worth noting that the equations appear to apply satisfactorily across the entire range of identified microstructural morphologies. In terms of practical significance, the proposed equations allow for the determination of the microstructural spacing for a given thermal parameter, whether it be Ṫ_L_ or V_L_. In other words, these equations allow for estimating the microstructural parameter based on a known thermal parameter, without the need for quantitative microstructural characterization.

**Figure 7:**
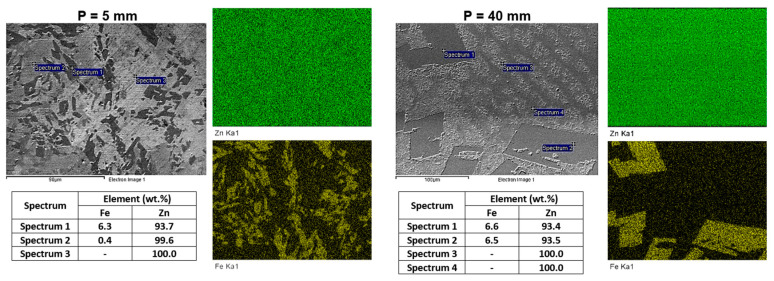
SEM/EDS analyses of Zn-2wt.%Fe alloy samples with different microstructural morphologies.

Figure 7 shows EDS point analysis and elemental mapping of SEM micrographs obtained from the Zn-2wt.%Fe alloy samples, considering two distinct microstructural morphologies observed at positions 5 and 40 mm from the base of the casting. The EDS analyses confirmed the presence of iron in the FeZn_13_ particles (Spectra 1 and 2 in both cases), while the matrix was composed exclusively of zinc (Spectrum 3), as expected due to the low solubility of Fe in Zn [22,23]. This behavior is in agreement with the literature, which indicates distinct phase segregation in this alloy, where Fe tends to form well-defined intermetallics without significantly dissolving in the Zn matrix [24]. The uniform composition of the matrix, composed practically of 100%Zn, regardless of the observed morphology, reflects the chemical stability of the Zn-Fe system, even under varying thermal solidification conditions.

For the sample P = 40 mm, which displayed a microstructural change in the zinc-rich matrix with a cellular morphology, the analyses showed that this morphological change did not affect the chemical composition of the matrix, which remained practically entirely Zn, as evidenced by the comparison between Spectra 3 and 4. This compositional consistency, despite morphological changes, suggests that cooling conditions impact the morphologies of the phases, without significantly altering the elemental segregation in the Zn-2wt.%Fe alloy.

**Figure 8:**
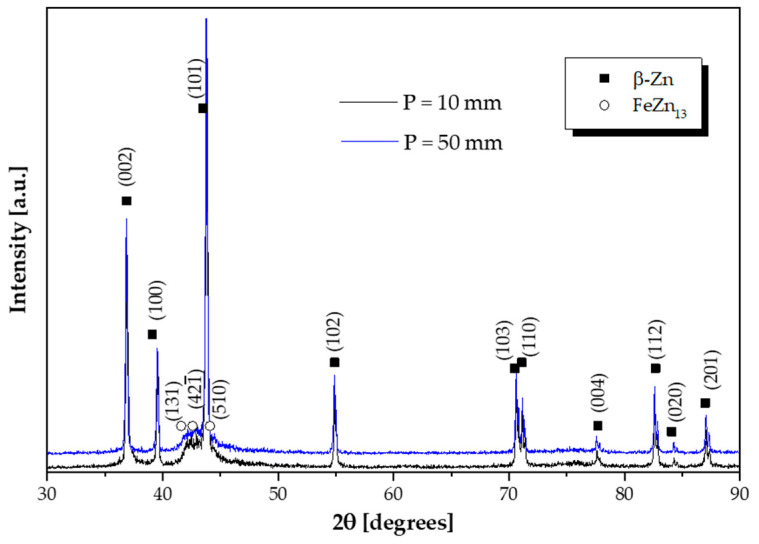
XRD patterns of the Zn-2wt.%Fe alloy for two distinct cooling conditions: P = 10 mm (rapid cooling) and P = 50 mm (slow cooling).

Figure 8 shows the XRD patterns of two samples from the Zn-2wt.%Fe alloy, representing distinct cooling conditions: one cooled slowly (P = 50 mm) and the other cooled rapidly (P = 10 mm). Both diffractograms corroborate the findings from previous SEM/EDS analyses (Figure 7), as well as comparisons with the literature and the Zn-Fe phase diagram. In both cases, only Zn and FeZn_13_ were the identified phases, confirming the consistency of the experimental results with the expected phase formation in this alloy. The presence of these phases is consistent with the low solubility of Fe in the Zn matrix [25], leading to the formation of FeZn_13_ particles at the contours of the Zn-rich matrix, as observed in the micrographs and elemental analyses.

**Figure 9:**
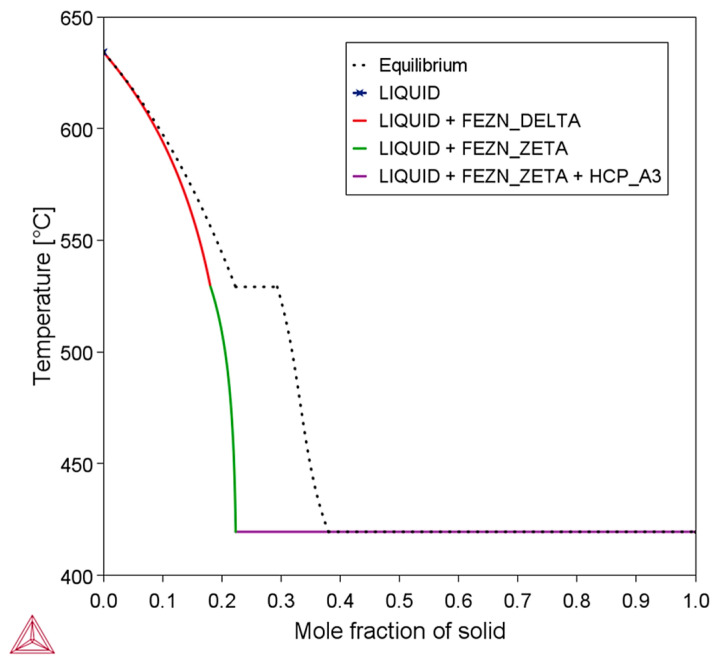
Scheil–Gulliver and equilibrium solidification paths for the Zn-2wt.%Fe alloy.

Figure 9 shows the Scheil–Gulliver and equilibrium solidification paths of the Zn-2wt.%Fe alloy, reinforcing that the final microstructure is composed of the Zn and FeZn_13_ phases. This graph was obtained using Thermo-Calc software version 2021b with its TCAL8 database (TCAL8: Al-Alloys v8.2 database). Considering that the alloy underwent several transformations during solidification, the final microstructure consisted of the HCP_A3 and FEZN_ZETA phases, corresponding to the Zn-rich matrix and the FeZn_13_ intermetallic, respectively. This correlation between theoretical predictions and experimental results reinforced the data obtained and the interpretation of microstructural phases formed during solidification under different cooling conditions.

### 3.4. Influence of Microstructure on Hardness

**Figure 10:**
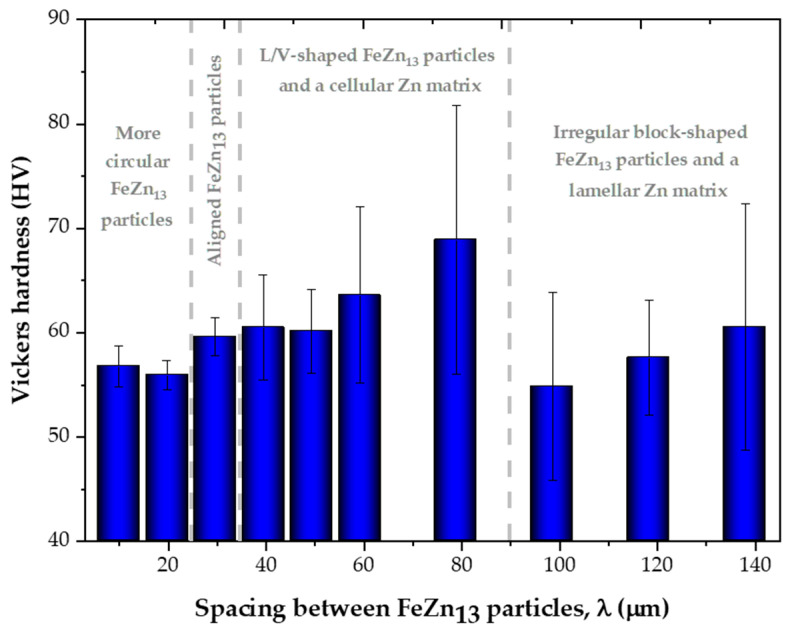
Hardness of the Zn-2wt.%Fe alloy as a function of the spacing between FeZn_13_ particles (λ). The dashed lines in the graph indicate the boundaries between different morphological patterns of the microstructural phases.

Figure 10 illustrates the relationship between the hardness of the Zn-2wt.%Fe alloy and the spacing between FeZn_13_ particles (λ). The variation in hardness can be explained by the occurrence of different microstructural morphologies. The observed changes in hardness correspond to the four distinct morphological patterns identified, indicating that transitions between these morphologies have a significant impact on the hardness of the alloy.

**Figure 11:**
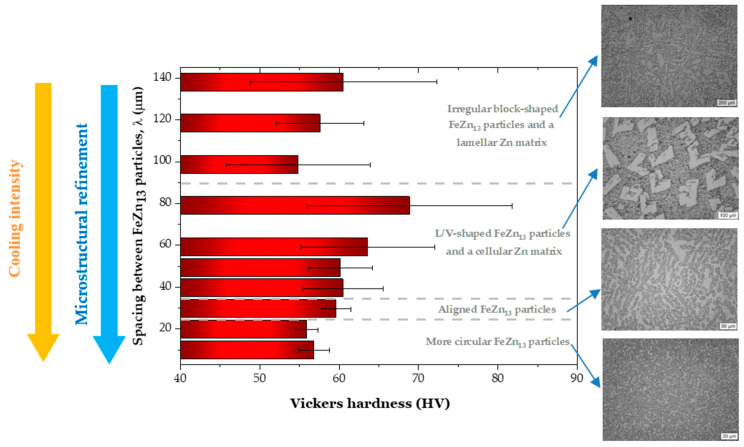
Main morphological changes in the microstructure of the Zn-2wt.%Fe alloy induced by varying cooling rates and their impacts on hardness. The dashed lines in the graph indicate the boundaries between different morphological patterns of the microstructural phases.

The hardness behavior contradicted the initial expectation that microstructural refinement, characterized by a reduction in the spacing between FeZn_13_ particles, would lead to a significant increase in the hardness of the Zn-2wt.%Fe alloy. Instead, the morphology and distribution of the intermetallic particles and the zinc-rich matrix seemed to have a more pronounced influence on hardness. At positions near the base of the casting (5 and 10 mm), intermetallic particles with slightly circular geometries were identified, explaining the low hardness levels in these regions. At the 15 mm position, the first microstructural transition occurred, marked by the alignment of FeZn_13_ particles into more organized patterns. Subsequently, L/V-shaped particles formed at the 20 mm position, which increased the average hardness, possibly due to the presence of sharp corners on the particles which accumulated local stresses. This profile continued up to the 40 mm position, maintaining a rough texture in the matrix and L/V-shaped FeZn_13_ particles, though with varying degrees of refinement, leading to hardness variations. At higher positions (40, 50, 60, and 70 mm), new intermetallic geometries and a predominance of more complex forms were identified, causing a slight decrease in hardness. Additionally, the Zn matrix developed a progressively more pronounced lamellar profile at higher casting heights. Figure 11 shows a summary of these observations.

The hardness was categorized according to the different morphologies observed. For the case of more circular FeZn_13_ particles (observed for Ṫ_L_ between 36.10 and 14.16 °C/s), the average hardness was 56.4 HV. For the case of aligned FeZn_13_ particles (observed for Ṫ_L_ = 8.19 °C/s), the average hardness was 59.6 HV. For the case of L/V-shaped FeZn_13_ particles with a cellular Zn matrix (observed for Ṫ_L_ between 5.55 and 2.18 °C/s), the average hardness was 63.3 HV. Lastly, for the case of irregular block-shaped FeZn_13_particles with a lamellar Zn matrix (observed for Ṫ_L_ between 1.61 and 1.02 °C/s), the average hardness was 57.7 HV. Thus, the hardness of Zn-2wt.%Fe can vary from 56.4 to 63.3 HV, depending on the morphologies of the microstructural phases. This range fell within the range reported in the literature for Zn-Fe alloy prepared by low-temperature sintering (48.5 to 64.6 HV) [13], Zn-0.8Fe alloy (about 60 HV) [26], and a directionally solidified Zn-1wt.%Mg alloy (65 HV) [27]. It is worth noting that the hardness related to the case of aligned FeZn_13_ particles (with an average hardness of 59.6 HV) was comparatively more similar to the hardness value of 60.1 HV reported for bone [28]. According to Li et al. [13], the increase in hardness can be attributed to the refined grain structure of the FeZn_13_ phase through the grain boundary pinning effect, where the FeZn_13_ phase acts as a barrier to grain growth. This is our first work with this alloy, and the finding that hardness is sensitive to the morphologies of the microstructural phases can open new perspectives for future research. We plan to explore mechanical behaviors beyond hardness, including tensile strength and fracture toughness, while also assessing the biodegradation performance and cytotoxicity of Zn-Fe alloys fabricated under different processing conditions. Understanding the correlation between microstructural features (especially the morphologies of the phases forming the microstructure) and in vivo performance would be important for tailoring Zn-Fe alloys for bioabsorbable implant applications.

## 4. Conclusions

The main objectives of this work, which were to investigate the microstructural evolution of the Zn-2wt.%Fe alloy and analyze its influence on hardness, were accomplished through a comprehensive experimental characterization of samples solidified under various cooling conditions. Considering the outcomes, the key conclusions are the following:

- The directional solidification experiment led to various cooling conditions along the length of the casting, allowing us to analyze the microstructural formation as a function of Ṫ_L_ and V_L_ ranges, which spanned from 1.02 to 36.10 °C/s and from 0.24 to 2.27 mm/s, respectively.
- The variations in the cooling conditions during solidification resulted in four different microstructural morphologies, corresponding to three morphological transitions as cooling decreased. The first transition caused the alignment of the FeZn_13_ particles. The second morphological transition involved the formation of FeZn_13_ particles with “V” or “L” profiles and the emergence of cellular morphology in the Zn-rich matrix. The third transition led to the development of a lamellar profile in the Zn-rich matrix.
- The morphologies of the microstructural phases were shown to play an important role in the hardness of Zn-2wt.%Fe, which could range from 56.4 to 63.3 HV, depending primarily on these morphologies. FeZn_13_ particles with slightly circular geometries favored low hardness levels. With the alignment of FeZn_13_ particles into more organized patterns, hardness slightly increased. V- and L-shaped FeZn_13_ particles increased the average hardness. FeZn_13_ particles with more complex forms, together with the Zn matrix, developed a progressively more pronounced lamellar profile, which induced a slight decrease in hardness.
- Identifying that hardness is sensitive to the microstructural phase morphologies of Zn-2wt.%Fe is a valuable insight for developing Zn-Fe alloys with improved properties. In terms of engineering applications, including bioabsorbable implants, the hardness values obtained here fall within the range reported in the literature.
- The outcomes of this work provide additional guidance for the development of Zn-Fe alloys and open new directions for future research, focusing on mechanical properties beyond hardness, such as tensile strength and fracture toughness, while also evaluating biodegradation and cytotoxicity under different processing conditions.

## Figures and Tables

**Table 1:** Chemical compositions [wt.%] of the metals used to prepare the Zn-based alloys.

Metals	Zn	Fe	Mn	Al	Ni	Cu	Mg	Pb
Zn	Balance	-	-	-	-	-	-	<0.1
Fe	-	Balance	-	-	0.01	0.01	-	-

## Data Availability

The raw data supporting the conclusions of this article will be made available by the authors on request.
